# A deep learning method for automatic segmentation of the bony orbit in MRI and CT images

**DOI:** 10.1038/s41598-021-93227-3

**Published:** 2021-07-01

**Authors:** Jared Hamwood, Beat Schmutz, Michael J. Collins, Mark C. Allenby, David Alonso-Caneiro

**Affiliations:** 1 Contact Lens and Visual Optics Laboratory, Centre for Vision and Eye Research, School of Optometry and Vision Science, Queensland University of Technology (QUT), Kelvin Grove, Qld 4059 Australia; 2 Centre in Regenerative Medicine, Institute of Health and Biomedical Innovation, Queensland University of Technology, Kelvin Grove, QLD 4059 Australia; 3 Metro North Hospital and Health Service, Jamieson Trauma Institute, Herston, QLD 4029 Australia; 4 Biofabrication and Tissue Morphology Laboratory, Centre for Biomedical Technologies, School of Mechanical Medical and Process Engineering, Queensland University of Technology (QUT), Herston, Qld 4000 Australia

**Keywords:** Biomedical engineering, Image processing, Computer science

## Abstract

This paper proposes a fully automatic method to segment the inner boundary of the bony orbit in two different image modalities: magnetic resonance imaging (MRI) and computed tomography (CT). The method, based on a deep learning architecture, uses two fully convolutional neural networks in series followed by a graph-search method to generate a boundary for the orbit. When compared to human performance for segmentation of both CT and MRI data, the proposed method achieves high Dice coefficients on both orbit and background, with scores of 0.813 and 0.975 in CT images and 0.930 and 0.995 in MRI images, showing a high degree of agreement with a manual segmentation by a human expert. Given the volumetric characteristics of these imaging modalities and the complexity and time-consuming nature of the segmentation of the orbital region in the human skull, it is often impractical to manually segment these images. Thus, the proposed method provides a valid clinical and research tool that performs similarly to the human observer.

## Introduction

**Figure 1:**
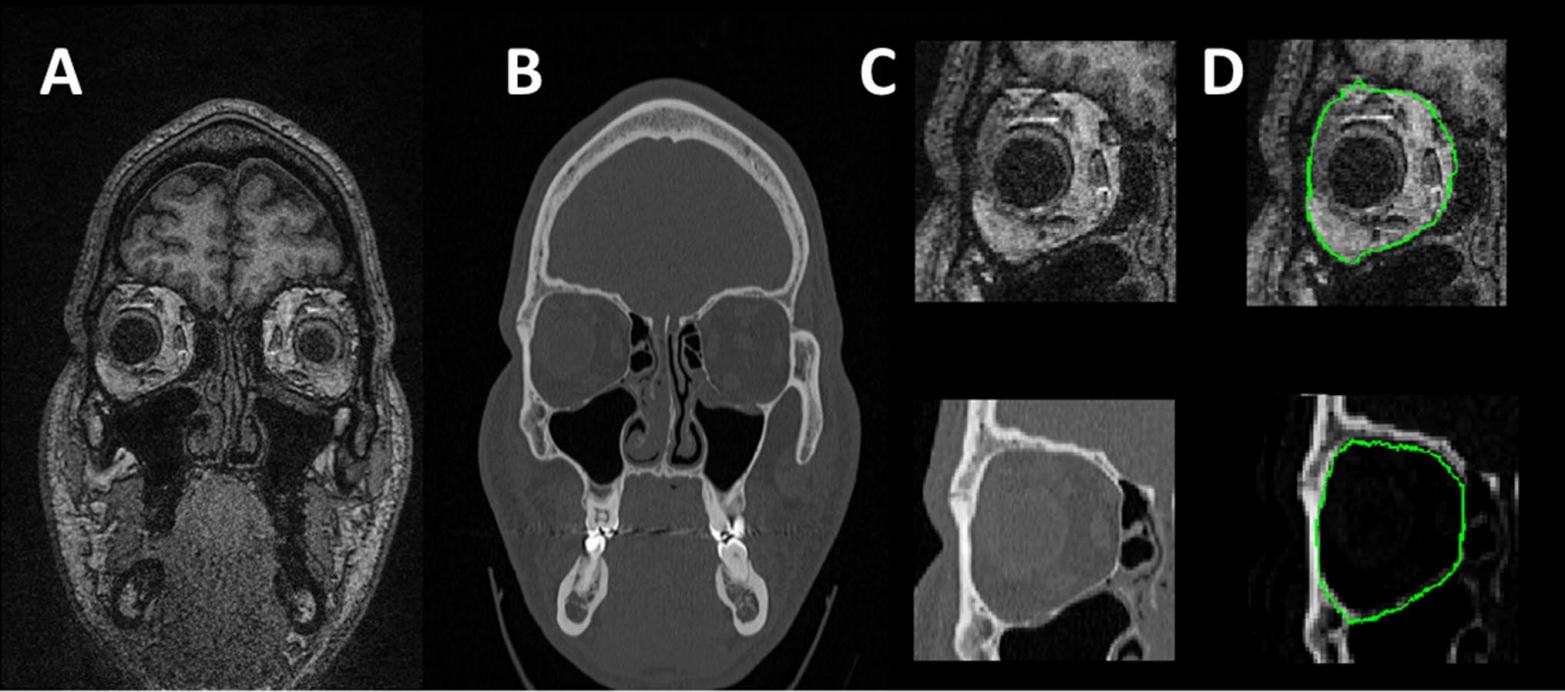
An example of a coronal plane image from the dataset for both T1-weighted 3 T MRI (**A**) and CT (**B**). Images belong to the same subject and represent approximately the same cross-section. A subplot of the region of interest (right orbit) for both imaging modalities is shown without (**C** upper panel MRI, lower panel CT) and with (**D** upper panel MRI, lower panel CT) the boundaries of the intraorbital bony contours in green.

The bony orbit (or eye socket) is the section of the skull containing the eyeball and surrounding ligaments and muscles. The orbit is not a single contiguous bone, instead consisting of seven bones and five openings. Clinically, the complex four-sided pyramidal configuration is divided into orbital roof, medial and lateral wall, and orbital floor^1^. The growth of the orbit correlates with ocular growth in foetuses^2^ and its shape is generally static in adults, with the exception of trauma such as fractures. This anatomical region can be imaged with magnetic resonance imaging (MRI)^3,4^, however computed tomography (CT) is the preferred modality for imaging^5,6^ because it is faster, cost effective, and provides better sensitivity to fractures^7^ which are the most common form of trauma associated with the orbit^3,8^. MRI and CT are both forms of non-invasive imaging that allow for measurement of internal structures in living subjects, and can be used to image the eye and surrounding ocular structures^7^. MRI uses magnetic fields to produce images, while CT uses ionising x-rays, hence the same structure will appear different in the corresponding images, even in scans of the same subject. Both modalities can be used to image a variety of structures, with MRI providing better imaging of soft-tissue, and CT providing better imaging of bones^3^. Acquisition times also differ, with MRI scans taking longer to acquire images^3^. Despite differences between the imaging modalities, the bony orbit region has been shown to be comparable when segmented in MRI and CT of the orbit. However, small but statistically significant shape differences were present between the MRI and CT volumes, with MRI models underestimating the size of orbit region when compared to the CT models^3^. Figure 1 shows an example of a cross-section CT and MRI image through a similar region of the bony orbit of the same subject, with the subplot showing a zoomed region with a delineation of the bony intraorbital surface.

Given the volumetric nature of these imaging modalities, segmentation of the complex orbital region in the human skull can be a time-consuming task. Manual segmentation and/or correction of CT images by an experienced clinician takes in the order of 30 min^1^, while segmentation from MRI can take up to 4 h for an intact orbit^3^ and 4–8 h for an orbit with fractures^9^. Thus, automatic or semi-automatic methods of segmentation are of great value for clinical and research applications. MRI and CT imaging modalities are widely used as diagnostic tools to assess ocular conditions, traumatic injuries, to visualize benign, malignant, and inflammatory processes in the orbit, as well as creating the basis for producing patient-specific physical models for pre-bending of orbital reconstruction plates^7,9–12^. Therefore it is important to develop methods for image segmentation, like those proposed in this study, since the structural information will support and complement clinical decision making.

In recent years, machine learning methods are commonly used for medical image analysis, especially for segmentation tasks, as they provide state of the art performance. Within the domain of medical image analysis, fully convolutional networks, a subtype of neural networks focused on working on the whole of a 2D cross-sectional image at once, are often used for segmentation or labelling tasks. Neural networks are commonly used for various tasks associated with MRI imaging, and show state of the art performance on a range of image analysis tasks including denoising^13,14^, super-resolution analysis of specific types of scans^15–17^, and synthesis of images for data augmentation^18^. Machine learning has been used for segmentation of MRI scans, with brain segmentation being a common application of these models^19–22^ as well as other soft tissues segmentation tasks such as the lungs^23^, abdominal organs^24^, or pelvic organs^25^. Similarly, a range of studies have proposed the use of machine learning in the analysis of CT images, where common applications include bone^26,27^ and organ segmentation^28,29^. Additionally, there has been research into image modality translation, converting MRI images to their equivalent CT representation, using neural networks^30^.

Broadly speaking, deep learning segmentation methods can be performed with fully convolutional networks or patch-based classification techniques. The former works on the entire image to make a per-pixel decision in a single run through the trained network, while the latter works on small sections of the image (patches) to predict the likelihood that this patch belong to a particular region (i.e. class) of interest. After this, the patch travels through the network to construct the prediction for the entire image. Fully convolutional networks take less time to process an image than the patch-based methods when tasked with whole image segmentation or labelling, and present comparable or superior performance in common accuracy metrics^31^. One of the most popular fully convolutional neural networks for image segmentation is U-Net^32^ which was proposed for use of in cell histology segmentation and has since been used in many other automatic methods for segmentation and labelling. U-Net has been used to localise and segment intervertebral discs in MRI images^33^ and to segment tissues in CT images such as livers and tumours^34,35^ or various organs in the chest^36^.

While commercial software applications (iPlan CMF: Brainlab AG, Munich, Germany; Bonelogic CMF Orbital: Disior, Helsinki, Finland) are available for the automated segmentation of the orbit from CT images, there does not appear to be any prior use of fully automatic methods for the segmentation of the orbit with MRI scans. At the time of writing, the reported studies used either fully manual segmentation, or combined manual segmentation with a thresholding based method, for delineating the bony orbital surface^3,37^. In this work, a novel two-stage deep learning segmentation method that extracts the intra-orbital bony contour in MRI and CT images is proposed. The proposed fully automatic method can be adapted to segment both imaging modalities, which allows to directly compare the network performance with MRI and CT images. The organization of the paper is as follows: “Methods” presents the proposed method and describes the data set and metrics for evaluation. “Results and discussion” compares the performance of the automated technique in the CT and MRI imaging modalities versus the manual segmentation by an experienced observer, while concluding remarks are provided in “Conclusion”.

## Methods

**Figure 2:**
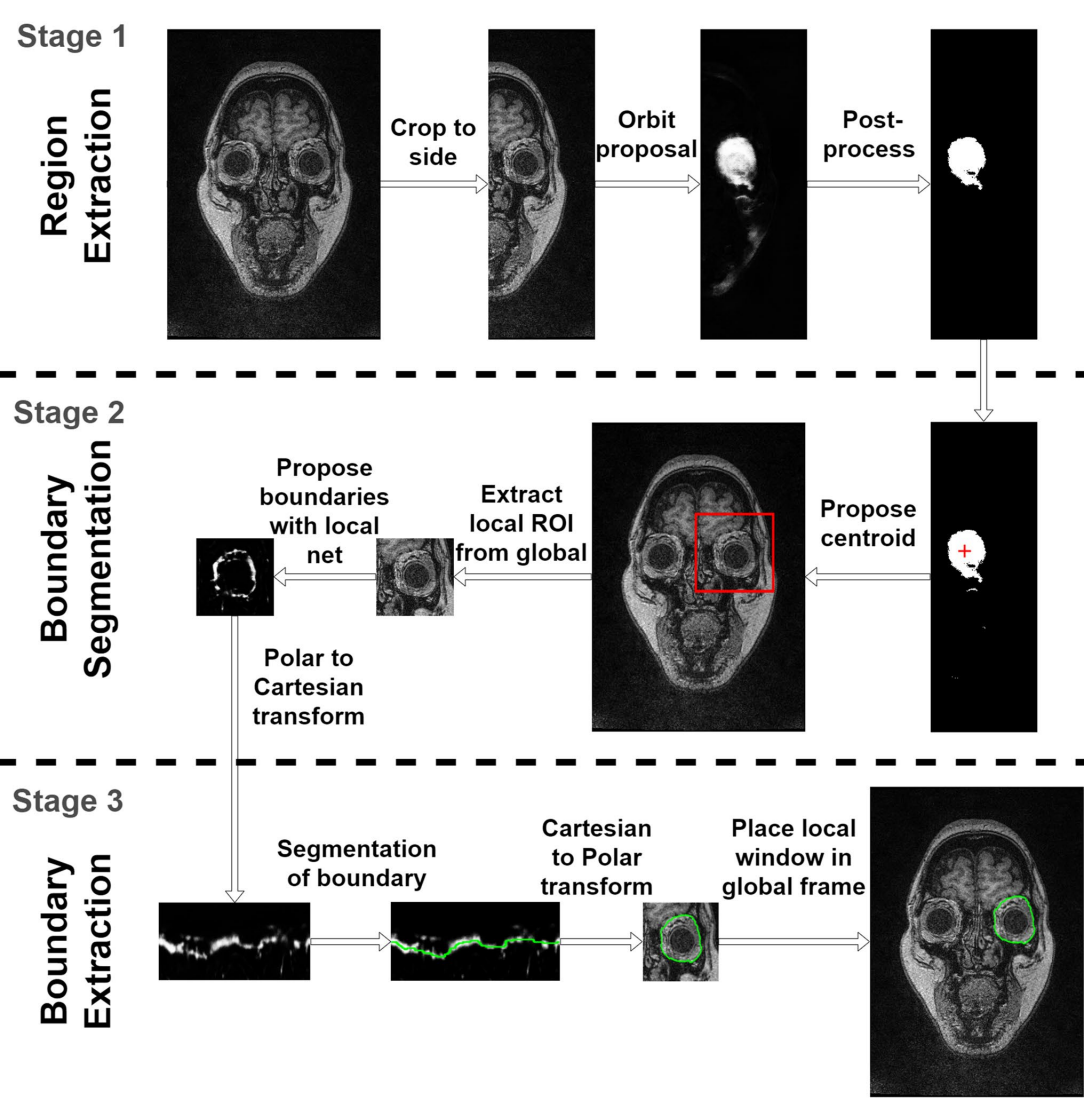
A diagram of the general image analysis process, with each row representing one of the three proposed stages. In order (top to bottom), the initial stage is the global extraction of the region of interest, followed by the local segmentation of the orbit boundary, and finally the boundary extraction. The first two stages use deep learning methods to produce a boundary likelihood map, while the final processing step use image processing techniques to extract the boundary.

A three-stage process is proposed for segmentation of the orbit and Fig. 2 provides an overview of the stages used to extract the boundary of interest. In the first stage, a full semantic neural network is used to localise a coarse estimate of the centroid of the orbit, which is later used for further analyses. This localisation is done at the image level by taking the centroid of all the largest single connected components after post-processing. The second step also uses a fully semantic neural network to extract the boundary probability map of the orbit in a local area of the image around the centroid proposed in stage one. The final stage is a segmentation of the proposed boundary within the probability map using a graph-search technique.

**Figure 3:**
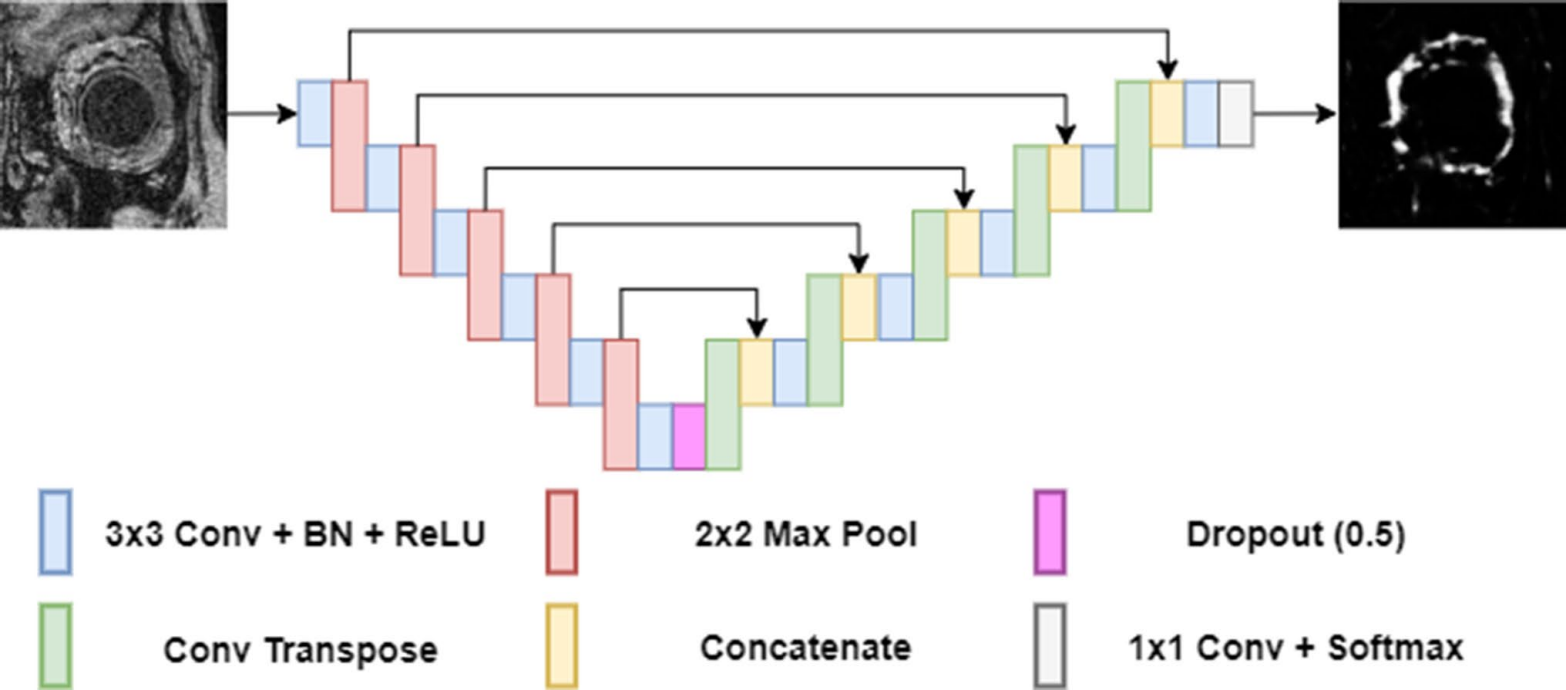
Diagram of the fully semantic neural network (U-net) used in for stages 1 and 2 of the method. An example of the MRI image for the stage 2 (boundary prediction task) is shown. The prediction maps use grey colour scale, where values close to white indicate a high likelihood of a boundary being present. Conv indicates convolutional layer, BN the batch normalization and ReLU the rectified linear activation unit.

In this work, two independent networks are used to maximise the performance of each step, although both networks could be trained simultaneously to both localise and segment, here the two networks are kept independent to separately optimise the performance for each task (this is shown in the results section). Stages one and two have the same network architecture, which is used for different purposes (the first stage performs a region location and the second stage performs a boundary segmentation). Thus, here the network is first introduced, before going into the specifics of the method. The network architecture used in this method is shown in Fig. 3. This neural network architecture uses a variant of a CNN called an “encoder-decoder” network. The encoder uses a number of convolutional layers for feature extraction and pooling layers for reducing spatial resolution and increasing context. The decoder is used to learn patterns and extract features to iteratively increase the spatial resolution and construct the output. For this study, an updated version of U-Net was used. The modification of the network includes the addition of batch norm layers^38^ in every encoder and decoder block, and a dropout layer^39^ at the bottleneck. Both batch norm and dropout are commonly used to improve neural network training and performance but were not included in the original U-Net implementation. Due to the fully convolutional nature of U-net, the same network can function on images of multiple sizes, provided the dimension of the original image can be pooled the required number of times.

### Stage 1: Global extraction of the orbit region

This initial step aims to localize the region of the orbit within the image. The ideal truth generated by this network would be a perfect labelling of the inner volume of the orbit, however in practice there is an uncertainty in the probability maps generated by this network. It is likely that the class imbalance in the data (i.e. boundary pixels represent a small portion of the whole image) may negatively impact the segmentation performance, even if the network is trained with a loss function that can handle data imbalance. Visual assessment of the network output demonstrates that in nearly all cases it manages to label the correct area, but the delineation tends to be coarse and not always to an acceptable quality for segmenting the orbit. Thus, this initial step is used to extract the region of the orbit. To aid in better localisation of the orbit, the output of this network is post-processed, first by filling all holes within the image, and then by discarding all but the largest single connected component. The centroid of this component is then calculated and used as the region’s proposed central value of the orbit for stage 1. The region extracted has constant dimensions of 128 pixels by 128 pixels in both imaging modalities.

The global network was trained with the Adam optimiser with a default initial learning rate of 0.001 and no learning rate change during training. The loss function is a cross-entropy loss and was trained for 100 epochs with the network with the best validation accuracy chosen as the final network, the mini-batch used for each training iteration was set to 128. The training data is shuffled before each training epoch.

### Stage 2: Segmentation of orbit boundary

Similar to the first step, the local network utilizes a modified version of the U-net architecture presented in Fig. 3. This network takes the local region of the image containing the orbit and generates a probability map where high values correspond to a pixel being a boundary pixel between the orbit and surrounding tissue. The truth generated by this network is a boundary rather than a filled-in shape, with the ideal results being a probability map that activates where the orbit transitions to the cavity. Unlike the global network, there is no post-processing of the network output.

The network is trained with different parameters to the global network. Specifically, a stochastic gradient descent with momentum, an initial learning rate of 0.01, and no learning rate change during training. The loss function is a cross-entropy loss, and a 500 epoch training time with the network, with the best validation accuracy chosen as the final network, the mini-batch used for each training iteration was set to 128. The training data is shuffled before each training epoch.

### Step 3: Boundary extraction

The third and final step of the method involves segmenting the boundary from the proposed prediction map estimated by the local network. The boundary is essential for generating virtual and physical 3D models, which facilitate preoperative planning, pre-bending of orbital reconstruction plates, surgical navigation, and postoperative assessment^3,9,11,40^. This provides important information for clinicians and researchers and can be used to plan surgical intervention, and 3D reconstruction of the tissue. The probability maps produced in Step 2 appear as a ring representing the location of the boundary of interest. Although a method such a circular Hough transform could be used to fit a circle to each slice of a scan, the orbit becomes irregular in shape (triangular) at smaller sizes (i.e. back of the eye) and this approach can fail or provide suboptimal region extraction. For the efficiency of using a single segmentation method for every scan within the volumetric acquisition, a graph-search segmentation was used. Given that a roughly circular boundary proves slightly difficult to segment by a graph-search, the image is transformed from Cartesian into Polar coordinates. This is similar to unwrapping the image around the centre and converts the ring shape into a curved line. The transformed image is segmented as a single path then the resulting boundary is transformed back from Polar to Cartesian coordinates.

To extract the path, a Dijkstra’s graph-search algorithm was used. This methods has been widely used and described in a range of ocular applications to extract boundary information^34,41^. In brief, the method finds the shortest path from one side of the image to the other. The pixel within the image representing nodes of the graph. The connection between nodes (pixels) is weighted inversely to the probability of them being a boundary, as shown by the equation below

$$W_{{ab}} = 2 - \left( {P_{a} + P_{b} } \right)~$$

where $W_{{ab}}$ is the weight of the edge between nodes (pixels), *a* and *b*, $P_{a}$ is the probability node *a* is a boundary, and $P_{b}$ is the probability node *b* is a boundary. The starting and finishing points of the path are automatically selected at opposite corners of the image. Two additional columns of pixels are added each side of the image with low weights. This encourages the graph-search to quickly connect with the path of interest and these additional columns are removed after the algorithm has terminated.

In practice, the segmentation has a tendency to vary in radius when nodes were connected diagonally, so the node (graph) connections were restricted to only horizontal (left to right) and vertical movements (up and down) in polar coordinates. This has the effect of reducing the node connections along solely radius or solely angular degree (polar) directions while not limiting connections along both radius and angular degree directions at the same time.

### Post-processing

Post-processing of the image is performed after the 3-step process is completed, with each image being averaged with its preceding and succeeding scans to prevent any outlying regions of confusion from decreasing performance. Given the ocular orbit is monotonically decreasing in width and height away from the medial slice, this serves to constrain any cases where a single under- or over-estimation would lower performance. Due to the lack of preceding and succeeding scans respectively, the first and last scans are omitted from the subsequent analysis.

### Data

A dataset that contains images from the two different scanning modalities (CT and MRI) from the same group of subjects was used to assess performance of the method. CT and MRI scans, available from a previous study^3^, were collected and the bony orbits were manually extracted from 11 subjects (9 male), with the patients mean age of 30 years. The CT dataset was retrieved from various routine clinical CT scanners that image the bony orbit, the data was acquired using a standard protocol of 120 kVp and a 0.5 to 1.0 mm slice thickness. The MRI was performed on a clinical 3-T MRI scanner (Trio Tim, Siemens, Erlangen, Germany) with a standard head coil to obtain adequate bone soft tissue contrast and the following specifications: T1-weighted multiplanar resolution sequence; repetition time of 2.030 ms, echo time of 4.16 ms, average of 1, frequency of 123.26 Hz, flip angle of 9 degree, pixels size 0.5 mm and slice thickness of 0.5 mm. This study was approved by the human research and ethics committees at the Royal Brisbane and Women’s Hospital and at the Queensland University of Technology. All participants were over 18 years old and signed an informed consent agreement. All participants were treated according to the tenets of the declaration of Helsinki. In this dataset, only a single bony orbit, which was free from fracture, was manually segmented within each scan and that half of the image was fed to the network for image analysis. It is worth noting that both image modalities produce a volumetric scan for each subject, however in this study the coronal images are treated as individual images for the purpose of processing and training the network. Thus, in this work the terms ‘image’ or ‘slice’ refer to a coronal slice of the volumetric data. Analysis is performed both for the individual image (slice of the volumetric scan) and the volumetric level for completeness.

All MRI scans had the same digital resolution of 352 × 512 pixel 16-bit tiff files, however the number of images per subject differed. All MRI scans were taken with a voxel size of 0.5 × 0.5 × 0.5 mm. The MRI data was broadly split into a training set of 6 subjects consisting of 366 scans and a testing set of 5 subjects consisting of 340 images with the orbit present. Images from CT scans were also collected as 16-bit tiff files, however the image size and resolution of the scans differed from subject to subject. The CT scan voxel sizes ranged between 0.3 mm to 0.51 mm in width, 0.3 mm to 1 mm in height, and all had an axial plane spacing of 0.5 mm. The full acquisition parameters for both datasets has been described previously^3^. The CT scans were resized, cropped, or interpolated as necessary to be of an identical resolution as the MRI (0.5 mm in all image planes) since standardization is necessary for performance comparisons across the different imaging modalities. The CT data was divided the same way as the MRI data, resulting in a training set of data from 6 subjects consisting of 443 scans, and a testing set of data from 5 subjects consisting of 363 scans.

For stage 1 and 2, Data augmentation techniques were included to improve data diversity. During training, images were reflected horizontally with 50% probability, and a + /- 10 degrees rotation angle was chosen randomly from a continuous uniform distribution to rotate the images. This enhances the diversity of the data and has a positive effect on the learning and performance of the network.

For both datasets, only frontal plane images with the orbit present as a closed contour were included in the final dataset. Thus, the orbit is treated in this paper as a bounding structure by closing off all openings. For the sake of comparing both methods, the split of the data (in terms of subjects) was the same across methods. The slightly different number of total images per group, is due to the different instruments’ image resolution.

In both cases, a K-fold cross-validation was used with k = 6 and each subject serving as the validation data set once, while keeping the testing set fixed. The training was performed for each imaging modality (MRI and CT) independently. For each fold, the training set contains 5 participants data and the validation set 1 participants data. In this way, all 6 participants data were used at least once within the validation. This technique uses multiple splits within the data to reduce the effects of randomness of the split^42^. This approach results in multiple trained models and allows us to also evaluate the effect of ensemble, which is the combination of multiple outputs of machine learning models to improve stability of the final prediction and it can also improve the overall performance of the model. The ensemble method uses majority voting, thus it takes the predictions from all networks and it computes the majority vote to provide a final boundary prediction map, and finally segments the single resulting prediction map.

Prior to input to either network, all image pixel intensities were normalised to fall between the range of 0 and 1 (both inclusive). Additionally, the CT images for the global network were pre-processed to increase the contrast of the image, this is done by saturating the bottom 1% and the top 1% of all pixel values, which reduces the effect of extreme outliers influencing the intensities of the input images.

### Metrics for image evaluation

Manually segmented images, available from a previous study^3^, were used as the ground truth for training as well as performance comparison. For the first stage of the method (extraction of the orbit region), the evaluation of performance was done by calculating the centroid errors by taking the Euclidean distance between the true centroid and proposed centroid (this metric is reported in pixels). For the second stage (boundary detection), the performance was compared by evaluating the Dice coefficient, also known as F1 score, for both background and orbit classes within the region extracted around the orbit (128 × 128 pixels). Background indicates any pixel that does not belong to the orbit boundary. This evaluation shows the accuracy for both positive predictive power and negative predictive power, which a 1-class Dice coefficient may fail to fully explain. Average Dice coefficients are calculated for both the per-slice (per-image) as well as for the entire volume.

$${\text{Dice}} = ~\frac{{2 \times T_{P} }}{{2 \times T_{P} + F_{P} + F_{N} }}$$

where $T_{P}$ is the number of true positives, $F_{P}$ is the number of false positives, and $F_{N}$ is the number of false negatives, all calculated in a per-pixel basis. Values for each fold, the mean of all folds, and the ensemble are presented. A boundary error calculation is performed by calculating the difference between the truth and prediction, this provides the boundary errors as the mean absolute error. Similarly, the Hausdorff distance (the greatest of all the distances from a point in one set to the closest point in the other set) was extracted as an additional metric to extract the boundary error.

## Results and discussion

**Figure 4:**
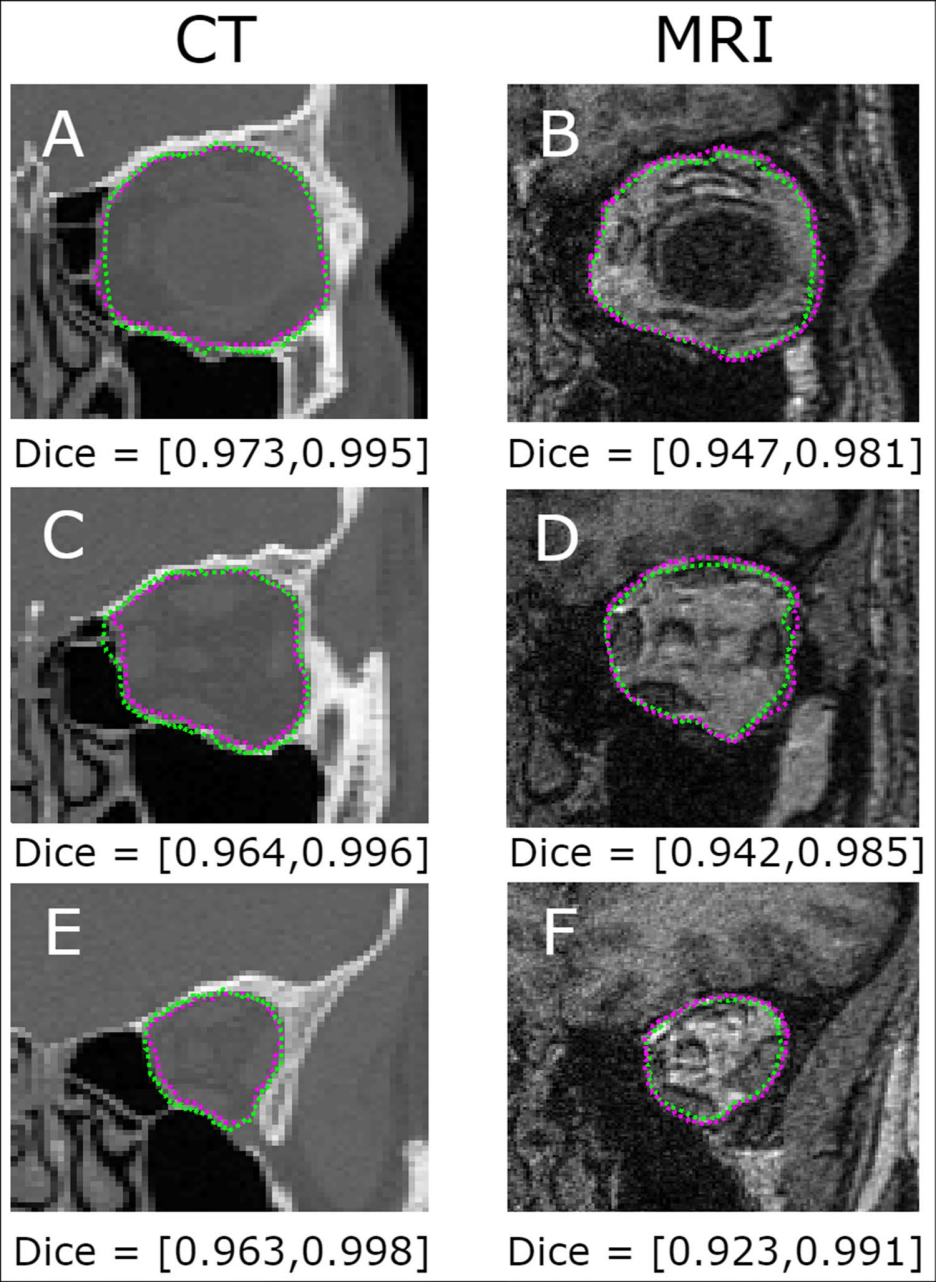
A visual comparison of the performance of the proposed method for examples from CT (left A, C and E) and T1-weighted 3 T MRI (right B, D and F) imaging modalities. Images belong to the same subject and represent approximately the same cross-section. The subplots show different cross-sections (depths) from the same orbital volume. Green dotted line represents the proposed automatic method and the dotted magenta is the ground truth. The dice include the orbit (left) and background (right) values.

This study explores the performance of a custom developed multi-step deep learning method to extract the orbit in two different imaging modalities. Given that the image appearance of MRI and CT image modalities is very different, the proposed method is applied to both imaging modalities separately, which should improve performance. Tables 1, 2 and 3 present the results for the MRI and CT datasets. Figure 4 shows some representative images to demonstrate the performance across the two imaging modalities. The technique shows differences in performance across the dataset, which are worth investigating. For the MRI dataset the ensemble performance did not show a clear improvement across the different Dice metrics in comparison to the mean of the individual folds. For this MRI dataset, the maximum volumetric Dice (best performance) is found in the individual fold with 0.917 for orbit and 0.989 for the background, or 0.825 and 0.987 if the per-slice Dice is considered (Table 2). For the CT dataset, the ensemble performance did show a significant improvement in performance when compared to the mean of the individual folds. The volumetric Dice ensemble performance is 0.930 for orbit and 0.995 for the background, or 0.887 and 0.995 if the per-slice Dice is considered (Table 3). The inferior values of the per-slice Dice compared to the volumetric Dice are due to the anatomical shape of the region. As the posterior orbit region becomes smaller, small pixel classification errors have a big impact on the Dice metric and subsequently on the per-slice Dice.

**Table 1:** Results summary with the mean ± standard deviation for centroid and boundary errors for the MRI and CT datasets.

Fold	MRI dataset		CT dataset	
	Centroid errors (pixels)	Boundary MAE (pixels)	Centroid Errors (pixels)	Boundary MAE (pixels)
1	18.69 ± 24.73	2.10 ± 0.67	4.00 ± 1.73	2.04 ± 0.93
2	19.47 ± 29.46	2.40 ± 0.52	6.02 ± 2.75	2.77 ± 1.45
3	15.72 ± 13.45	2.85 ± 1.59	5.45 ± 0.43	1.39 ± 0.64
4	18.24 ± 25.74	1.68 ± 0.34	2.04 ± 1.11	1.07 ± 0.30
5	11.87 ± 15.60	1.91 ± 0.31	8.27 ± 3.00	2.17 ± 0.80
6	23.64 ± 31.89	2.26 ± 1.24	2.36 ± 1.55	1.06 ± 0.41
Fold Mean	17.93 ± 23.47	2.20 ± 0.78	4.69 ± 1.76	1.75 ± 0.76
Ensemble	20.43 ± 26.48	2.45 ± 0.74	3.05 ± 1.42	1.20 ± 0.53

MAE indicates the mean absolute error. The values for the individual folds are presented along with their mean and the ensemble (based on majority voting).

**Table 2:** Results summary with the mean ± standard deviation for volumetric and per-slice Dice for the MRI dataset.

Fold	Vol. dice (bony orbit)	Vol. dice (background)	Per-slice dice (bony orbit)	Per-slice dice (background)
1	0.878 ± 0.052	0.983 ± 0.007	0.781 ± 0.133	0.982 ± 0.014
2	0.889 ± 0.016	0.985 ± 0.002	0.771 ± 0.147	0.986 ± 0.010
3	0.912 ± 0.052	0.988 ± 0.007	0.789 ± 0.095	0.986 ± 0.007
4	0.880 ± 0.027	0.984 ± 0.003	0.795 ± 0.147	0.984 ± 0.015
5	0.917 ± 0.027	0.989 ± 0.003	0.825 ± 0.067	0.987 ± 0.008
6	0.881 ± 0.056	0.984 ± 0.007	0.789 ± 0.166	0.983 ± 0.015
Fold Mean	0.893 ± 0.078	0.986 ± 0.010	0.791 ± 0.126	0.985 ± 0.012
Ensemble	0.813 ± 0.069	0.975 ± 0.008	0.768 ± 0.160	0.982 ± 0.011

The values for the individual folds are presented along with their mean and the ensemble (based on majority voting).

**Table 3:** Results summary with the mean ± standard deviation for volumetric and per-slice Dice for the CT dataset.

Fold	Vol. dice (bony orbit)	Vol. dice (background)	Per-slice dice (bony orbit)	Per-slice dice (background)
1	0.888 ± 0.052	0.992 ± 0.004	0.812 ± 0.089	0.992 ± 0.004
2	0.782 ± 0.154	0.983 ± 0.013	0.761 ± 0.081	0.982 ± 0.014
3	0.911 ± 0.033	0.994 ± 0.002	0.847 ± 0.076	0.994 ± 0.002
4	0.927 ± 0.023	0.995 ± 0.002	0.883 ± 0.060	0.995 ± 0.002
5	0.841 ± 0.088	0.987 ± 0.008	0.749 ± 0.093	0.987 ± 0.008
6	0.930 ± 0.029	0.995 ± 0.002	0.887 ± 0.069	0.995 ± 0.002
Fold mean	0.887 ± 0.058	0.991 ± 0.004	0.842 ± 0.071	0.991 ± 0.004
Ensemble	0.930 ± 0.032	0.995 ± 0.002	0.864 ± 0.090	0.995 ± 0.002

The values for the individual folds are presented along with their mean and the ensemble (based on majority voting).

When comparing the method for the two imaging modalities, the CT results show a slightly superior performance to the MRI for the bony orbit, and marginally superior for the background. This superior performance with the CT is not surprising since this instrument provides a clearer imaging of the bony region of interest, showing a greater contrast between background and bone that greatly facilitates the segmentation process. This superior performance with the CT data is also observed in the initial centroid detection which shows an error of 20 pixels for the MRI and 3.05 for the CT. Considering the square ROI size of 128 pixels, this corresponds to a 15% and 3% error in the centroid localization for the MRI and CT respectively. Similarly, when calculating the boundary error, the method used with the MRI data shows slightly higher absolute errors (MAE = 2.45) than with the CT data (MAE = 1.20) in the ensemble model.

For individual folds, a negative correlation between centroid error and orbit Volumetric Dice was found, with r^2^ = − 0.81 and r^2^ = − 0.85 for the MRI and CT dataset, respectively. Thus, indicating that the localisation network (stage 1) may have an impact on the subsequent output of the centroid position computed from stage 2 (centroid accuracy).

To assess the effect on performance of a single stage versus the 2-stage method, and to demonstrate the importance of localizing the area of interest, an additional analysis was performed on two different versions of the dataset. The first dataset contained half of the image (to avoid the detection of the unlabelled eye), while the second dataset contained images that were cropped on a region of interest around the label. Both used the ensemble mode and the same training specifications as stage 2. Across the testing dataset the mean Dice coefficient improved by 10% and over 30% for the MRI and CT dataset respectively when segmenting the second dataset with the smaller regions of interest. The use of 2-stage architecture has also been applied in other ophthalmic studies to segment the regions of interest^43,44^.

Additionally, to compare the performance of the model versus other deep learning methods, the proposed 2-stage method was used, substituting the U-net model for a different deep learning architecture. Particularly three models were tested, all are based on a DeepLab v3 + convolutional neural network for semantic image segmentation with different pretrained models, including ResNet 18, MobileNet v2 and ResNet 50. To facilitate comparison, results are provided only for the final ensemble model (Table 4). Similar to the previous results, the overall Dice metrics showed higher values for the CT than the MRI dataset. For the CT, the proposed method showed a sightly superior performance to the ResNet 18 and the MobileNet v2, while the difference is larger with the ResNet 50, which shows the worst performance. Similar for the MRI dataset, the ResNet 50 performance was worse than the other models. The difference from ResNet 18 or MobileNet v2 with the proposed model are small, with the proposed method outperforming ResNet 18 and MobileNet v2 in three out of the four reported metrics. The poorer performance of ResNet 50 across both imaging modalities may be associated with the ResNet 50 model’s complexity. ResNet 50 has 23 million trainable parameters while ResNet 18 and MobileNet v2 have 11 and 3.4 million parameters respectively. A larger image dataset may be needed to train and improve ResNet 50 performance.

**Table 4:** Results summary with the mean ± standard deviation for volumetric and per-slice Dice scores for the CT and MRI datasets.

Method	Vol. dice (bony orbit)	Vol. dice (background)	Per-slice dice (bony orbit)	Per-slice dice (background)
**CT**				
Proposed	0.930 ± 0.032	0.995 ± 0.002	0.864 ± 0.090	0.995 ± 0.002
ResNet 18*	0.913 ± 0.029	0.986 ± 0.002	0.840 ± 0.094	0.970 ± 0.002
MobileNet v2*	0.911 ± 0.032	0.987 ± 0.001	0.845 ± 0.089	0.972 ± 0.002
ResNet 50*	0.886 ± 0.039	0.964 ± 0.002	0.828 ± 0.102	0.937 ± 0.003
**MRI**				
Proposed	0.813 ± 0.069	0.975 ± 0.008	0.768 ± 0.160	0.982 ± 0.011
ResNet 18*	0.822 ± 0.058	0.934 ± 0.012	0.709 ± 0.277	0.924 ± 0.013
MobileNet v2*	0.855 ± 0.041	0.947 ± 0.009	0.730 ± 0.232	0.959 ± 0.012
ResNet 50*	0.818 ± 0.057	0.936 ± 0.011	0.721 ± 0.236	0.917 ± 0.013

The values represent the final ensemble deep learning model and are based on majority voting. Models with an asterisk (*) are based on a DeepLab v3 + convolutional neural network for semantic image segmentation with a specific pretrained model.

Table 5 provides a summary of the results while assessing the Hausdorff distance (the greatest of all the distances from a point in one set to the closest point in the other set, in here the ‘set’ represents the segmentation points). Similar to overall metrics, a superior performance (lower distances) can be observed for the CT dataset, which is expected since the orbital boundary is better defined in this imaging modality. The difference across the different tested models is only marginal. For the MRI dataset, the differences are more pronounced with the proposed model and MobileNet v2 showing a superior performance. Based on the comparison with different deep learning methods, the proposed technique based on the U-net network seems to compare favourably for the image segmentation tasks, providing comparable or sightly superior performance across the reported metrics. However, other DL architectures may also be applicable to this image segmentation task.

**Table 5:** Results summary with the mean ± standard deviation for the Hausdorff distance for the CT and MRI datasets.

Method	CT (pixels)	MRI (pixels)
Proposed	3.121 ± 1.442	4.737 ± 2.600
ResNet 18*	2.933 ± 1.123	6.443 ± 2.925
MobileNet v2*	3.113 ± 1.205	4.933 ± 2.260
ResNet 50*	3.549 ± 1.250	7.173 ± 2.966

The values represent the final ensemble deep learning model and are based on majority voting. Models with an asterisk (*) are based on a DeepLab v3 + convolutional neural network for semantic image segmentation with a specific pretrained model.

Since we do not have access to the implementation of previously published methods or access to previously used datasets, comparison with results from the literature is limited. The results are not directly comparable with the findings of clinical studies as they typically report orbital segmentation accuracy in terms of surface-based deviations or orbital volumes. The only comparable study^1^, reports a mean Dice coefficient of 0.881 ± 0.035 for the automated model-based segmentation of orbital volumes from clinical cone beam CT (CBCT) scans, which has slightly lower performance than the proposed method. Using the same automated model-based method for the segmentation of orbital volumes from CBCT scans of 30 patients, Wagner et al. 2016 found no significant differences between manually segmented (ground truth) and automatically segmented orbital volumes^45^. As Dice coefficients of the proposed are only slightly higher, this would suggest that the accuracy of the presented method is in the clinically acceptable range. The obtained MAEs appear to confirm this. While the MRI dimensional MAE of 1.2 mm is double that of the corresponding CT error of 0.6 mm, both are below the acceptable 2 mm tolerance for surgical reconstruction of the orbital bony anatomy^46^.

In terms of processing time, the proposed method can fully segment the orbit in an entire volumetric scan in around 1.5 min for the MRI dataset and 3 min for the CT dataset. While commercial software applications can segment an entire orbit from CT within the same timeframe, the proposed method presents a significant step forward for the automated segmentation of MRI data. Even in its present form, the method could augment manual segmentation of the orbit from MRI, which should significantly reduce the current manual segmentation times which can be up to 4 h^3^. The prohibitively long segmentation times of the orbit from MRI compared to CT are currently the limiting factor for MRI to be routinely used as a first-line imaging modality for orbital trauma^3,9,37^, which would otherwise be attractive due to its non-ionising radiation.

A limitation of the proposed method is that it does not generalise to segment the orbit’s entrance as the data was modelled as a closed boundary. The hyperbolic paraboloid shape of the orbital entrance^47^ results in discontinuous boundaries of the bony anatomy in the frontal plane images. For the same reason, segmentation of a fractured orbit in MRI and CT images is not yet feasible with this method. We intend to include the segmentation of the orbit’s entrance as a future extension to the method, however, certain aspects of the process may have to be changed.

Despite the promising results, the small available training dataset is another limitation in the current study that may affect performance. It is reasonable to expect that a larger dataset would improve the accuracy of the method. While considering the dataset, it is worth noting that MRI images contain a lot more detail (i.e. tissue detail) than the CT, which have a more uniform feature distribution (Fig. 4). This may further emphasize the importance of having extra data for model training, particularly in the case of MRI. Exploring deep learning strategies that can generate synthetic medical images such as data augmentation techniques^48^, or generative adversarial networks^49,50^, may be required.

## Conclusion

This paper presents a novel method for automatically segmenting the bony orbit within scans from two clinical imaging modalities (MRI and CT). The proposed method is an end-to-end two-stage deep framework that first localizes the region of interest, followed by a fully semantic segmentation on this region of interest and shows a high level of agreement with human manual segmentation across both modalities. The method is also significantly faster than manual segmentation, with a single volume fully segmented in around one and a half minutes for the MRI, and three minutes for the CT, while an expert may take hours to manually segment the same volume for a single subject. The performance demonstrated that the proposed method provides a tool that can be useful for both clinical and research purposes.

## Data Availability

The datasets analysed during the current study are currently not publicly available. However, the algorithms developed in this work are available from the corresponding author on reasonable request.

## References

[1] Becker, M., Friese, K., Wolter, F., Gellrich, N. & Essig, H. Development of a reliable method for orbit segmentation & measuring. In *2015 IEEE International Symposium on Medical Measurements and Applications (MeMeA) Proceedings* 285–290.

[2] Velasco-Annis C (2015). Normative biometrics for fetal ocular growth using volumetric MRI reconstruction. Prenat. Diagn..

[3] Schmutz B (2014). Magnetic resonance imaging: An accurate, radiation-free, alternative to computed tomography for the primary imaging and three-dimensional reconstruction of the bony orbit. J. Oral Maxillofac. Surg..

[4] Goldberg RA, Relan A, Hoenig J (1999). Relationship of the eye to the bony orbit, with clinical correlations. Aust. N. Z. J. Ophthalmol..

[5] Kubal WS (2008). Imaging of orbital trauma. Radiographics.

[6] Lee H-J, Jilani M, Frohman L, Baker S (2004). CT of orbital trauma. Emerg. Radiol..

[7] Lin KY, Ngai P, Echegoyen JC, Tao JP (2012). Imaging in orbital trauma. Saudi J. Ophthalmol..

[8] Chang EW, Manolidis S (2005). Orbital floor fracture management. Facial Plast. Surg..

[9] Cooper T, Schmutz B, Hsu E, Lynham A (2020). Magnetic resonance imaging for three-dimensional printing of the bony orbit: Is clinical use imminent?. Int. J. Oral Maxillofac. Surg..

[10] LeBedis CA, Sakai O (2008). Nontraumatic orbital conditions: Diagnosis with CT and MR imaging in the emergent setting. Radiographics.

[11] Metzger MC (2006). Individual preformed titanium meshes for orbital fractures. Oral Surg. Oral Med. Oral Pathol. Oral Radiol. Endodontol..

[12] Simon GJB (2005). Rethinking orbital imaging: Establishing guidelines for interpreting orbital imaging studies and evaluating their predictive value in patients with orbital tumors. Ophthalmology.

[13] Bermudez C (2018). Learning implicit brain MRI manifolds with deep learning. Proc. SPIE Int. Soc. Opt. Eng..

[14] Benou A, Veksler R, Friedman A, Riklin Raviv T (2017). Ensemble of expert deep neural networks for spatio-temporal denoising of contrast-enhanced MRI sequences. Med. Image Anal..

[15] Zeng K (2018). Simultaneous single- and multi-contrast super-resolution for brain MRI images based on a convolutional neural network. Comput. Biol. Med..

[16] Liu C (2018). Fusing multi-scale information in convolution network for MR image super-resolution reconstruction. Biomed. Eng. Online.

[17] Chaudhari AS (2018). Super-resolution musculoskeletal MRI using deep learning. Magn. Reson. Med..

[18] Shin H-C, Gooya A (2018). Simulation and Synthesis in Medical Imaging.

[19] García-Lorenzo D, Francis S, Narayanan S, Arnold DL, Collins DL (2013). Review of automatic segmentation methods of multiple sclerosis white matter lesions on conventional magnetic resonance imaging. Med. Image Anal..

[20] Smistad E, Falch TL, Bozorgi M, Elster AC, Lindseth F (2015). Medical image segmentation on GPUs: A comprehensive review. Med. Image Anal..

[21] Dora L, Agrawal A, Panda R, Abraham A (2017). State-of-the-Art methods for brain tissue segmentation: A review. IEEE Rev. Biomed. Eng..

[22] Lundervold AS, Lundervold A (2019). An overview of deep learning in medical imaging focusing on MRI. Z. Med. Phys..

[23] Kovacs W (2017). Holistic segmentation of the lung in cine MRI. J. Med. Imaging (Bellingham).

[24] Bobo MF (2018). Fully convolutional neural networks improve abdominal organ segmentation. Proc. SPIE Med. Imaging.

[25] Lei Y (2020). Male pelvic multi-organ segmentation aided by CBCT-based synthetic MRI. Phys. Med. Biol..

[26] Lindgren Belal S (2019). Deep learning for segmentation of 49 selected bones in CT scans: First step in automated PET/CT-based 3D quantification of skeletal metastases. Eur. J. Radiol..

[27] Kim YJ, Ganbold B, Kim KG (2020). Web-based spine segmentation using deep learning in computed tomography images. Healthc. Inform. Res..

[28] Ait Skourt B, El Hassani A, Majda A (2018). Lung CT image segmentation using deep neural networks. Procedia Comput. Sci..

[29] Ibragimov B, Xing L (2017). Segmentation of organs-at-risks in head and neck CT images using convolutional neural networks. Med. Phys..

[30] Wolterink JM, Tsaftaris SA, Gooya A, Frangi AF, Prince JL (2018). Simulation and Synthesis in Medical Imaging.

[31] Kugelman J (2019). Automatic choroidal segmentation in OCT images using supervised deep learning methods. Sci. Rep..

[32] Ronneberger, O., Fischer, P. and Brox, T. U-net: Convolutional networks for biomedical image segmentation. In *International Conference on Medical image computing and computer-assisted intervention* 234–241 (2015).

[33] Dolz J, Desrosiers C, Ben Ayed I, Zheng D, Belavy D, Cai Y, Li S (2020). Computational Methods and Clinical Applications for Spine Imaging.

[34] Kugelman J, Alonso-Caneiro D, Read SA, Vincent SJ, Collins MJ (2018). Automatic segmentation of OCT retinal boundaries using recurrent neural networks and graph search. Biomed. Opt. Express.

[35] Jin Q, Meng Z-P, Sun C, Wei L, Su R (2018). RA-UNet: A hybrid deep attention-aware network to extract liver and tumor in CT scans. Front. Bioeng. Biotechnol..

[36] Haleem A, Javaid M (2018). Role of CT and MRI in the design and development of orthopaedic model using additive manufacturing. J. Clin. Orthop. Trauma.

[37] Eley KA, Watt-Smith SR, Golding SJ (2017). “Black Bone” MRI: A novel imaging technique for 3D printing. Dentomaxillofac. Radiol..

[38] Ioffe, S. and Szegedy, C., Batch normalization: Accelerating deep network training by reducing internal covariate shift. In *International conference on machine learning* 448–456 (2015).

[39] Srivastava N, Hinton G, Krizhevsky A, Sutskever I, Salakhutdinov R (2014). Dropout: A simple way to prevent neural networks from overfitting. J. Mach. Learn. Res..

[40] Schmelzeisen R (2004). Navigation-aided reconstruction of medial orbital wall and floor contour in cranio-maxillofacial reconstruction. Injury.

[41] Chiu SJ (2010). Automatic segmentation of seven retinal layers in SDOCT images congruent with expert manual segmentation. Opt. Express.

[42] Liu Y, Chen P-HC, Krause J, Peng L (2019). How to read articles that use machine learning: Users’ Guides to the Medical Literature. JAMA.

[43] Bian X, Luo X, Wang C, Liu W, Lin X (2020). Optic disc and optic cup segmentation based on anatomy guided cascade network. Comput. Methods Prog. Biomed..

[44] Lu, D. *et al.* Cascaded Deep Neural Networks for Retinal Layer Segmentation of Optical Coherence Tomography with Fluid Presence. *arXiv preprint* (2019).

[45] Wagner MEH (2016). Model-based segmentation in orbital volume measurement with cone beam computed tomography and evaluation against current concepts. Int. J. Comput. Assist. Radiol. Surg..

[46] Scolozzi P, Jaques B (2008). Computer-aided volume measurement of posttraumatic orbits reconstructed with AO titanium mesh plates: Accuracy and reliability. Ophthalmic Plast. Reconstr. Surg..

[47] Osaki TH (2013). Comparison of methodologies in volumetric orbitometry. Ophthalmic Plast. Reconstr. Surg..

[48] Mikołajczyk, A. and Grochowski, M. Data augmentation for improving deep learning in image classification problem. *In 2018 international interdisciplinary PhD workshop (IIPhDW)* 117–122 (2018).

[49] Kugelman, J. *et al.* Constructing synthetic chorio-retinal patches using generative adversarial networks. *In 2019 Digital Image Computing: Techniques and Applications* 1–8 (2019)

[50] Kugelman, J. *et al.* Data augmentation for patch-based OCT chorio-retinal segmentation using generative adversarial networks. *Neural Comput. Appl.* 1–16 (2021).

